# Unlocking the hidden potential of Mexican teosinte seeds: revealing plant growth-promoting bacterial and fungal biocontrol agents

**DOI:** 10.3389/fpls.2023.1247814

**Published:** 2023-10-04

**Authors:** Esaú De-la-Vega-Camarillo, Juan Alfredo Hernández-García, Lourdes Villa-Tanaca, César Hernández-Rodríguez

**Affiliations:** Laboratorio de Biología Molecular de Bacterias y Levaduras, Departamento de Microbiología, Escuela Nacional de Ciencias Biológicas, Instituto Politécnico Nacional, Ciudad de México, Mexico

**Keywords:** teosinte, massive sequencing, next generation sequencing (NGS), maize, bacteriome

## Abstract

The bacterial component of plant holobiont maintains valuable interactions that contribute to plants’ growth, adaptation, stress tolerance, and antagonism to some phytopathogens. Teosinte is the grass plant recognized as the progenitor of modern maize, domesticated by pre-Hispanic civilizations around 9,000 years ago. Three teosinte species are recognized: *Zea diploperennis*, *Zea perennis*, and *Zea mays*. In this work, the bacterial diversity of three species of Mexican teosinte seeds was explored by massive sequencing of 16S rRNA amplicons. *Streptomyces*, *Acinetobacter*, *Olivibacter*, *Erwinia*, *Bacillus*, *Pseudomonas*, *Cellvibrio*, *Achromobacter*, *Devosia*, *Lysobacter*, *Sphingopyxis*, *Stenotrophomonas*, *Ochrobactrum*, *Delftia*, *Lactobacillus*, among others, were the bacterial genera mainly represented. The bacterial alpha diversity in the seeds of *Z. diploperennis* was the highest, while the alpha diversity in *Z. mays* subsp. mexicana race was the lowest observed among the species and races. The Mexican teosintes analyzed had a core bacteriome of 38 bacterial genera, including several recognized plant growth promoters or fungal biocontrol agents such as *Agrobacterium*, *Burkholderia*, *Erwinia*, *Lactobacillus*, *Ochrobactrum*, *Paenibacillus*, *Pseudomonas*, *Sphingomonas*, *Streptomyces*, among other. Metabolic inference analysis by PICRUSt2 of bacterial genera showed several pathways related to plant growth promotion (PGP), biological control, and environmental adaptation. The implications of these findings are far-reaching, as they highlight the existence of an exceptional bacterial germplasm reservoir teeming with potential plant growth promotion bacteria (PGPB). This reserve holds the key to cultivating innovative bioinoculants and formidable fungal antagonistic strains, thereby paving the way for a more sustainable and eco-friendly approach to agriculture. Embracing these novel NGS-based techniques and understanding the profound impact of the vertical transference of microorganisms from seeds could revolutionize the future of agriculture and develop a new era of symbiotic harmony between plants and microbes.

## 1 Introduction

The domestication of plants has played a crucial role in the cultural and economic advancement of societies across the globe. Through domestication, humanity has cultivated plants that provide several benefits, including food, beverages, medicine, raw materials for industry, and even elements that have cultural or social significance (Milla et al., 2015; Purugganan, 2019).

The biological origin, diversification, and domestication of maize occurred in Mesoamerica, located in the center of Mexico. This grass of the Poaceae family had a seminal role in the origin, extension of agriculture, and culture of pre-Hispanic civilizations (Smith et al., 1981). One of the species of actual teosintes, *Zea mays* subsp. parviglumis, is the progenitor of all derivative *Zea mays* subsp. mays modern races. The human-driven domestication that started around 9,000 years ago is one of the most critical events in the history of agriculture (Doebley, 2004; Piperno et al., 2009; Sahoo et al., 2021).

Numerous groups of bacteria and fungi establish interactions with plants. It has been discovered that the overall health of plants is closely associated with the specific composition of microorganisms present both in the soil and the plants themselves (Gherbi et al., 2008; Miyambo et al., 2016; van der Heijden and Hartmann, 2016; Dastogeer et al., 2020). Plants maintain associations with microorganisms both outside and within their tissues. Endophytic microorganisms within the root, stem, leaves, flowers, and seeds maintain mutualistic symbiosis with the plant host (Frey-Klett et al., 2011; Mishra et al., 2015; De Mandal et al., 2021). Seed endophyte microorganisms can be transferred vertically to plant offspring, ensuring their permanence in favorable environments (Johnston-Monje & Raizada, 2011).

Few studies of culturable fractions of teosinte bacteria have been performed. Nitrogen-fixing *Paraburkholderia tropica* (formerly *Burkholderia tropica*) was isolated from the rhizosphere and stem of teosinte (Caballero-Mellado et al., 2004; Reis et al., 2004). Although this species has not been reported again associated with teosinte, other species and strains isolated from maize and sugarcane express plant growth promotion (PGP) and antifungal phenotypic features (Tenorio-Salgado et al., 2013; Bernabeu et al., 2018; Schlemper et al., 2018; Kuramae et al., 2020; Vio et al., 2022). Also, endophytic *Bacillus*, *Enterobacter*, *Methylobacterium*, and *Pantoea*, with variable PGP features, were repeatedly isolated from three different teosinte species (Johnston-Monje & Raizada, 2011). *Paenibacillus polymyxa* and *Citrobacter* sp. obtained from the same teosinte seeds inhibited fungal growth and mycotoxin production and maintained a potential to combat phytopathogens (Mousa et al., 2015). Currently, an important research topic is to elucidate how much of a plant’s phenotype, adaptive capacities, evolution, and productivity are due to its endospheric and rhizospheric microbiome (Santoyo et al., 2017; Kaur et al., 2021).

In that sense, next-generation sequencing (NGS) technologies have revolutionized the field of microbiology and have become an essential tool for studying the plant holobiont, which encompasses the plant and all its associated microorganisms. Identifying microbial species or microbiomes present in the plant holobiont is the first step to studying the complexity of the existing symbiosis (Simon et al., 2019; Marco et al., 2022).

In this work, the bacteriome of seeds of three teosinte species was explored by NGS of 16S rRNA gene. The alpha and beta diversities of bacterial genera, the core bacteriome of the teosinte species, and metabolic prediction of the main bacteria were documented. Many previously potential PGPB associated with maize were detected in teosintes. This work may lead efforts to isolate the cultivable fraction of these plant species that may be a reservoir of PGPB for use as biofertilizers and for biocontrol.

## 2 Materials and methods

### 2.1 Biological samples

**Figure 1:**
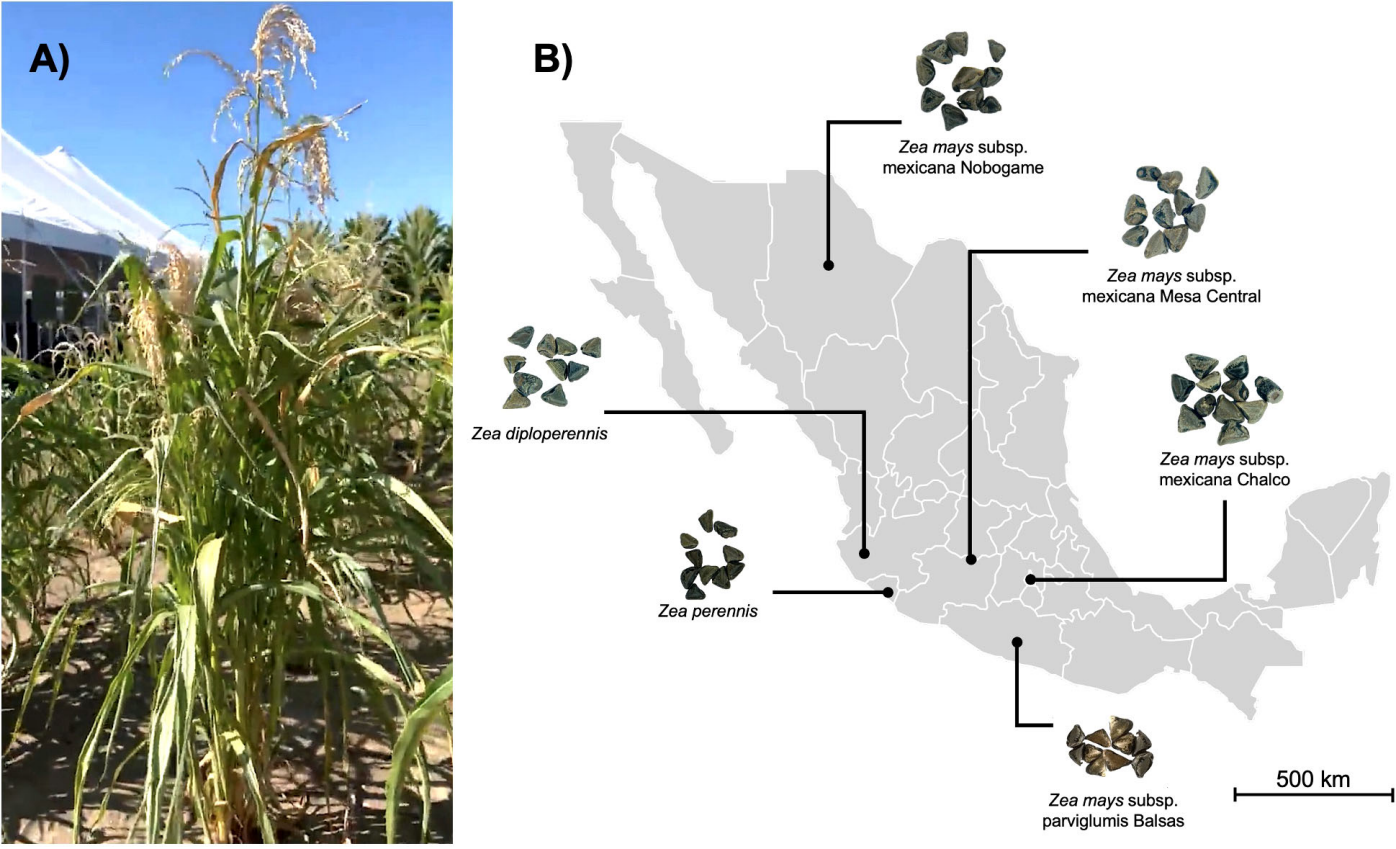
Teosinte in Mexico. **(A)** Mature teosinte plant, growing wild in maize fields. **(B)** Map of the distribution of the different races of teosintes in Mexico from which the samples were obtained.

Seeds of 6 different species, subspecies, and races of Mexican teosintes were used in this work: *Zea perennis*, *Zea diploperennis*, *Zea mays* subsp. mexicana race Nobogame, *Zea mays* subsp. mexicana race Mesa Central, *Zea mays* subsp. mexicana race Chalco and *Zea mays* subsp. parviglumis race Balsas. Teosinte seeds were provided by the International Maize and Wheat Improvement Center (CIMMYT) (Texcoco, Mexico). Information and access numbers for CIMMYT collections are presented in **Figure 1**; **Table 1**.

**Table 1:** Readings obtained and quality filtration from the massive sequencing of the 16S rRNA gene of teosinte seeds.

Teosinte specie	Top Name	Access Number	Location	Year of collection	Tissue	Total readings	Valid readings	ASV count
*Zea mays* subsp. *mexicana* race Nobogame	W.S.T. 92-2	CIMMYTMA 13572	Río Neva, Chihuahua 28.787993, -106.149427	2015	Seed	163,493	107,942	7,845
*Zea mays* subsp. *mexicana* race Chalco	MGB-CI 4	CIMMYTMA 29062	Tenango del Aire, México 19.173577, -98.853118	2015	Seed	147,015	93,799	3,985
*Zea mays* subsp. *mexicana* race Mesa Central	W.S.T. 92-4	CIMMYTMA 13574	Cuitzeo, Michoacán 19.982905, -101.171815	2015	Seed	189,400	133,559	6,986
*Zea mays* subsp*. parviglumis* race Balsas	K 67-5	CIMMYTMA 8755	Mazatlán, Guerrero 17.445471, -99.474217	2015	Seed	173,013	114,559	4,507
*Zea perennis*	MGB-CI 50	CIMMYTMA 29739	Coquimatlán, Colima 19.218588, -103.936109	2015	Seed	174,531	110,923	8,193
*Zea diploperennis*	LAS OYAS	CIMMYTMA 9476	Cuautitlán de García Barragán Jalisco 19.617700, -104.197447	2015	Seed	164,462	123,998	9,913

For later analyses the number of readings were rarefied to 93,799 readings (readings from the sample with the lowest number).

### 2.2 DNA extraction and 16S rRNA metabarcoding sequencing

Teosinte seeds (3 groups of 20 seeds per species) were washed with sterile distilled water for 48 h. The wash water was decanted, and the seeds were soaked in 5% sodium hypochlorite for 10 min and washed five times with sterile distilled water for 1 min. Finally, the seeds were disinfected with 95% ethyl alcohol for 10 min and washed five times with sterile distilled water for 1 min.

Three groups of 20 seeds for each variety were used for DNA extraction; later, these extractions per variety were pulled and sequenced. The extraction of metagenomic DNA was performed using the cetyltrimethylammonium bromide (CTAB) technique (Aboul-Maaty and Oraby, 2019). Primers 341F (5-Clamp 1-CCTACGGGAGGCAGCAG-3)/806R (5-ATTACCGCGGCTGCTGG-3) were used to amplify the V3-V4 regions of the 16S rRNA gene of the pulled metagenomic DNA obtained (Yang et al., 2017). A single 6-nucleotide label was added to the 5′ end of the initiators to distinguish PCR products. All PCR amplifications were performed in 30 μL reaction volumes containing 15 μL of 2 Phusion Master Mix (New England Biolabs, Ipswich, MA, USA), 0.2 μM of each forward and reverse primers, and 10 ng of metagenomic DNA. The thermal cycle conditions were: initial denaturation at 98°C for 1 minute followed by 30 cycles of 95°C for 10 sec, 50°C for 30 sec, and 72°C for 30 sec, with a final extension at 72°C for 5 min.

The amplification products were separated by 2% agarose gel electrophoresis (p/v) and purified with a GeneJET Gel extraction kit (Thermo Fisher Scientific, Waltham, MA, USA). Purified PCR products were sequenced on the Illumina HiSeq 2000 platform at Novogene Bioinformatics Technology Co. Ltd. (Beijing, China).

### 2.3 NGS analyses

FastQC performed the quality control for high throughput sequence data was performed by FastQC Version 0.12.0 (Andrews et al., 2010). The low-quality reads (Phred quality score < 25) and sequences <200 or > 500 bp long, containing ambiguous characters, homopolymers >6 bp, and mismatches in primers > 14 were removed from subsequent analyses (Lawley and Tannock, 2017).

Sequencing data were analyzed using the QIIME2™ software package (Bolyen et al., 2019). Sequences were quality-filtered, trimmed, denoised, and merged using DADA2 plugin (Callahan et al., 2016). Chimeric sequences, singletons, and doubletons were detected and removed by the DADA2 workflow. Representative ASVs were aligned with MAFFT and used for phylogenetic reconstruction in FastTree using plugin alignment and phylogeny (Faith and Baker, 2006). A trained Naïve Bayes classifier-based SILVA database (https://www.arb-silva.de/documentation/release-132/) was applied to assign the taxonomy (Agnihortry et al., 2020; Kõljalg et al., 2020). ASVs that could not be taxonomically identified were manually checked by performing BLAST searches in RDP (http://rdp.cme.msu.edu/) (Bacci et al., 2015) based on similarity thresholds for family, genus, and species at >90, >95, and >97%, respectively (Rosselló-Móra et al., 2017).

### 2.4 Alpha diversity analysis

The microbial diversity and microbial communities’ composition analyses were estimated with a series of scripts from QIIME2, including generating rarefied amplicon sequence variant (ASV) tables. To calculate α-diversity within these communities in all samples, the species richness was estimated using the observed ASV number and Chao1 (Chao, 1984), species diversity with Shannon (Shannon, 1948), and the dominance with Simpson index (Simpson, 1949) in QIIME2. The diversity indices of the samples were compared using the Mann-Whitney U test to evaluate the statistical significance between the samples (P < 0.05). Good’s coverage estimator was used to calculate the sequence coverage obtained for the 16S rRNA region datasets (Good, 1953).

### 2.5 Beta diversity analysis

The β-diversity comparison of seed bacteria among teosinte species was performed using UniFrac distances (Lozupone et al., 2011), both unweighted (phylogenetic richness) and weighted (relative abundance and phylogenetic richness) in MEGAN 6.21 software (Bağcı et al., 2019). Also, the Bray-Curtis dissimilarity was estimated using PAST 4.03 software (Hammer et al., 2001). Significant differences among bacterial communities of teosinte species were tested with the Monte Carlo method and Adonis test for UniFrac distances and the Bray-Curtis index, respectively. A Principal Coordinates Analysis (PCoA) to explore multidimensional patterns of diversity variation of bacterial communities among teosinte species was performed using unweighted and weighted UniFrac distances in PAST 4.03 (Hammer et al., 2001).

### 2.6 Visualization of diversity and abundance of samples and core bacteriome

The visualization, analysis, comparison, and contrast of the information of the ASV tables, heat-map graphs of relative abundance, and taxonomic co-occurrence analysis were made with MEGAN 6.21 (Bağcı et al., 2019) and TBtools v1.108 tools (Chen et al., 2020). The cut-off to define the core bacteriome of ASV in teosinte samples was a strict core of 100% (Bağcı et al., 2019).

### 2.7 Prediction of functional profiling of teosinte seed endophytic bacteria

The predictive functional profile of the endophytic bacterial communities of different teosinte seeds was inferred using the Phylogenetic Investigation of Communities by Reconstruction of Unobserved States 2 (PICRUSt2) software (Langille et al., 2013; Douglas et al., 2020) through the web application Galaxy7 and employing KEGG database (Afgan et al., 2016). The accuracy of metagenome predictions was determined with the nearest sequence-weighted taxon index (NSTI) that summarizes the extent to which microorganisms in a sample are related to sequence genomes, and they represent the average branch length that separates each ASV in a sample from a reference bacterial genome, weighting their relative abundance in each sample. Low values of this index indicate a closer mean relationship.

## 3 Results

### 3.1 Data quality analysis

The DNA sequence quality trimming was performed. **Table 1** summarizes the sample data and the number of trimmed DNA sequence data, showcasing only the high-quality, validated readings that met the predetermined quality criteria. The valid readings that oscillate between 93,799 and 133,559 are shown. The number of readings was reduced to the lowest value for subsequent analysis.

### 3.2 Bacterial communities’ analysis

The analysis of diversity to estimate richness and abundance in individual samples was carried out using multiple methods, as shown in **Table 2**. The samples of *Z. diploperennis* had the highest number of observed bacterial ASV (1822), while *Z. perennis* y *Z. mays* subsp. *parviglumis* teosinte harbored the greatest bacterial diversity estimated with the Simpson (0.0024) and reciprocal Simpson (3.8025) and Shannon (4.0142) indexes, respectively.

**Table 2:** Comparison of alpha diversity indices among teosinte races.

Teosinte specie	Chao1	Observed ASVs	Simpson index	Reciprocal Simpson index	Shannon	Goods coverage
*Zea mays* subsp. *mexicana* race Nobogame	1314.4	1187	0.0028	3.3652	3.0650	0.9978
*Zea mays* subsp. *mexicana* race Chalco	1163.7	964	0.0054	5.2839	3.5692	0.9979
*Zea mays* subsp. *mexicana* race Mesa Central	1732.9	1487	0.0033	4.9489	4.0020	0.9972
*Zea mays* subsp*. parviglumis* race Balsas	1955.2	1812	0.0032	5.8791	4.0142	0.9973
*Zea perennis*	1764.8	1525	0.0024	3.8025	3.5965	0.9969
*Zea diploperennis*	2011.2	1822	0.0029	5.3956	3.9023	0.9969

The α-diversity indices showed that *Z. diploperennis*, *Z. perennis*, and *Z. mays* subsp. *parviglumis* harbored higher diverse bacterial communities than *Z. mays subsp*. *mexicana* races Chalco, Nobogame, and Mesa Central. Moreover, the utilization of weighted UniFrac in β-diversity analysis unveiled that the estimated species turnover demonstrates the grouping of *Z. diploperennis*, *Z. perennis*, and *Z. mays* subsp. *parviglumis* within a single clade, while the races of *Z. mays* subsp. *mexicana* exhibit distribution in a separate clade.

**Figure 2:**
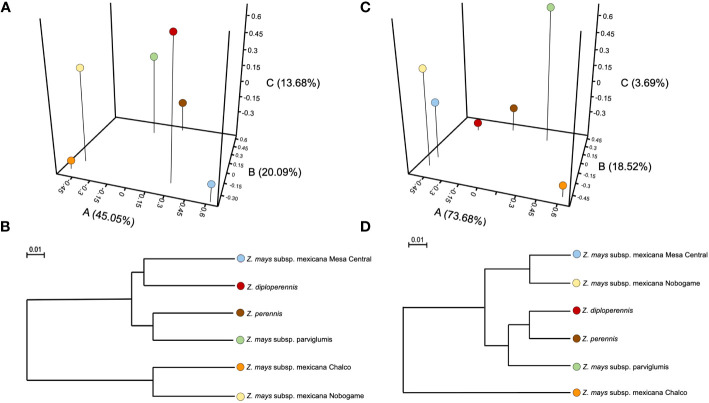
Principal coordinate analysis (PCoA) of β−bacterial diversity across all samples using unweighted **(A, B)** and weighted **(C, D)** UniFrac distances. Unweighted PCoA and UniFrac were performed to compare taxonomic groups assigned from massive sequencing of the 16S rRNA gene of seeds of different teosinte species and races.

The PCoA was performed using unweighted and weighted UniFrac distances and explained 78.6% (PCoA- 45.0%; PCoB-20.0%; PCoC- 13.6%) (**Figure 2A**) and 95.7% (PCoA: 73.6%; PCoB-18.5%; PCoC-3.6%) (**Figure 2C**) of the total bacterial genus-level variation, respectively. The unweighted PCoA showed that the bacterial diversity was different (P<0.05) among communities of teosinte races. However, in a weighted PCoA analysis, a rearrangement arose in the relationship among the different teosinte races according to bacterial communities’ diversity and abundance. *Z. mays* subsp. mexicana races Nobogame and Mesa Central were the most similar between them, followed by *Z. perennis* and *Z. diploperennis* pair, with his analysis does not show a clear grouping between the races of the species *Zea mays* (**Figures 2B–D**).

**Figure 3:**
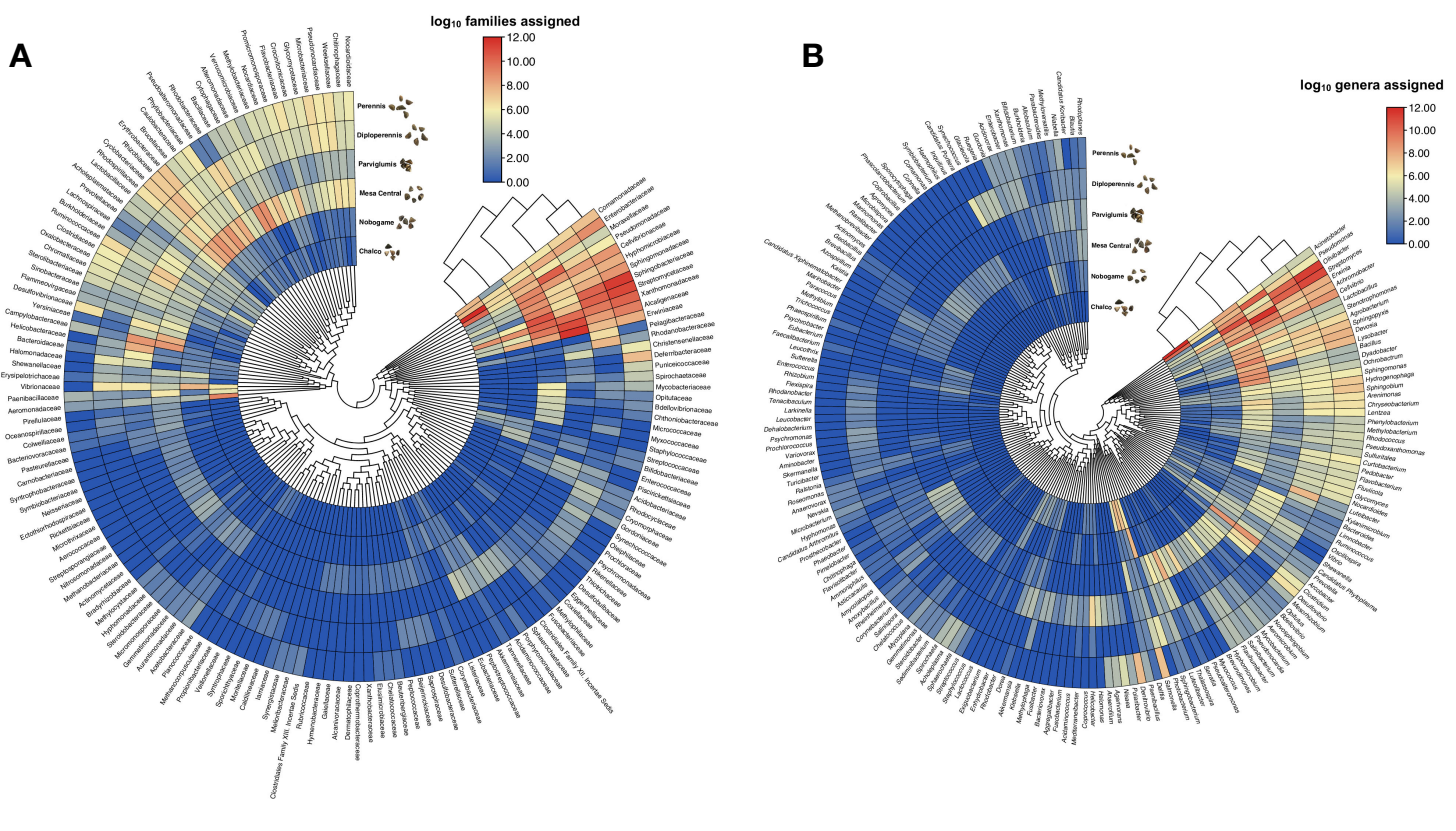
Endophytic bacterial diversity in Mexican teosinte seeds. **(A)** the relative abundance of families in teosinte seeds is expressed as the log_10_ of the total assigned readings, and **(B)** the relative abundance of bacterial genera is shown as the log_10_ of the total readings assigned. The bar color gradient represents high (red) and low (blue) readings.

Bacterial communities in the seeds of three teosinte species were remarkably diverse and consisted of 39 phyla and about 342 families with at least 1% abundance in samples (**Figure 3A**). The teosinte seed endophytes exhibited a dominant presence of Proteobacteria (8-40%) across all samples, highlighting its prominence as the most abundant phylum. Notably, the relative abundance analysis revealed several prominent bacterial families, including Enterobacteriaceae (0.6-3.9%), Vibrionaceae (0.2-1.7%), Xanthomonadaceae (3.2-8.5%), Aeromonadaceae (0.1-2.2%), Comamonadaceae (0.1-1.7%), Moraxellaceae (0.2-28.7%), Pseudomonadaceae (0.5-10.1%), Cyclobacteriaceae (0.4-2.4%), Cytophagaceae (0.2-3.9%), Sphingobacteriaceae (0.6-19.6%), Hyphomicrobiaceae (0.1-5.1%), Rhizobiaceae (0.1-3.6%), Rhosdospirilaceae (0.3-1.4%), Alcaligeneaceae (0.2-15.2%), Bacillaceae (0.1-3.8%), Lactobacillaceae (0.1-3.6%), Clostridiaceae (0.3-5.1%), Heliobacteriaceae (0.1-5.7%), Rhodobiaceae (0.3-6.1%), Rhodobacteriaceae (0.2-3.7%), and Ruminococcaceae (0.1-1.2%). A total of 572 genera were assigned, and the most abundant were *Streptomyces, Acinetobacter, Olivibacter, Erwinia, Bacillus, Pseudomonas, Cellvibrio, Achromobacter, Devosia, Lysobacter, Agrobacterium, Sphingopyxis, Stenotrophomonas, Ochrobactrum, Delftia*, and *Lactobacillus*. *Streptomyces* was the most abundant genera associated with *Z. mays* subsp. mexicana Mesa Central (17.7%) and *Z. diploperennis* (21.8%), for *Z. mays* subsp. mexicana Nobogame (19.5%) and *Z. mays* subsp. parviglumis (40.3%) was *Erwinia*, for *Z. perennis* was *Olivibacter* (17.9%), and for *Z. mays* subsp. mexicana Chalco was *Acinetobacter* (64.2%) (**Figure 3B**).

**Figure 4:**
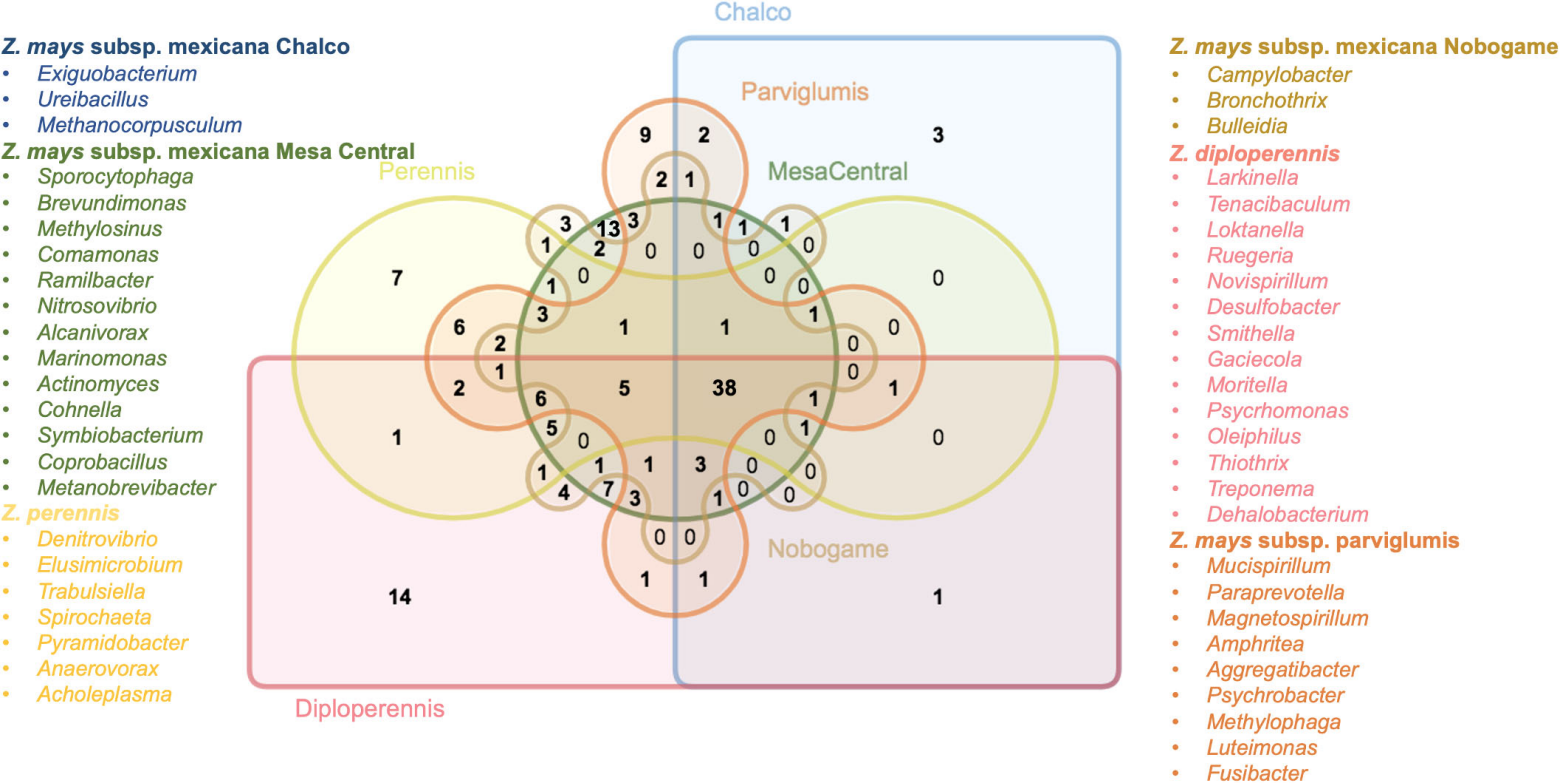
Comparison of bacterial genera in teosinte samples. Venn diagram showing the grouping relationships of microbial genera in the different species of teosinte (central bacteriome and accessory bacteriomes).

In the initial analysis, the distribution patterns of bacterial genera were examined within each teosinte race. The results indicated that varying numbers of bacterial genera exclusively associated with each race. *Z. mays* subsp. mexicana Chalco and *Z. mays* subsp. mexicana Nobogame exhibited three exclusive bacterial genera, while *Z. perennis*, *Z. mays* subsp. mexicana parviglumis, *Z. mays* subsp. mexicana Mesa Central, and *Z. diploperennis* showed seven, nine, thirteen, and fourteen exclusive bacterial genera, respectively (**Figure 4**). Although these findings could suggest the presence of bacterial genus-specific relationships within each teosinte race, the experimental design does not allow reaching that conclusion. Further investigations, such as metagenomic sequencing or functional profiling of the associated bacterial communities, and an extensive sampling would provide a more comprehensive understanding of the specific bacteriome and its potential implications for teosinte races.

**Figure 5:**
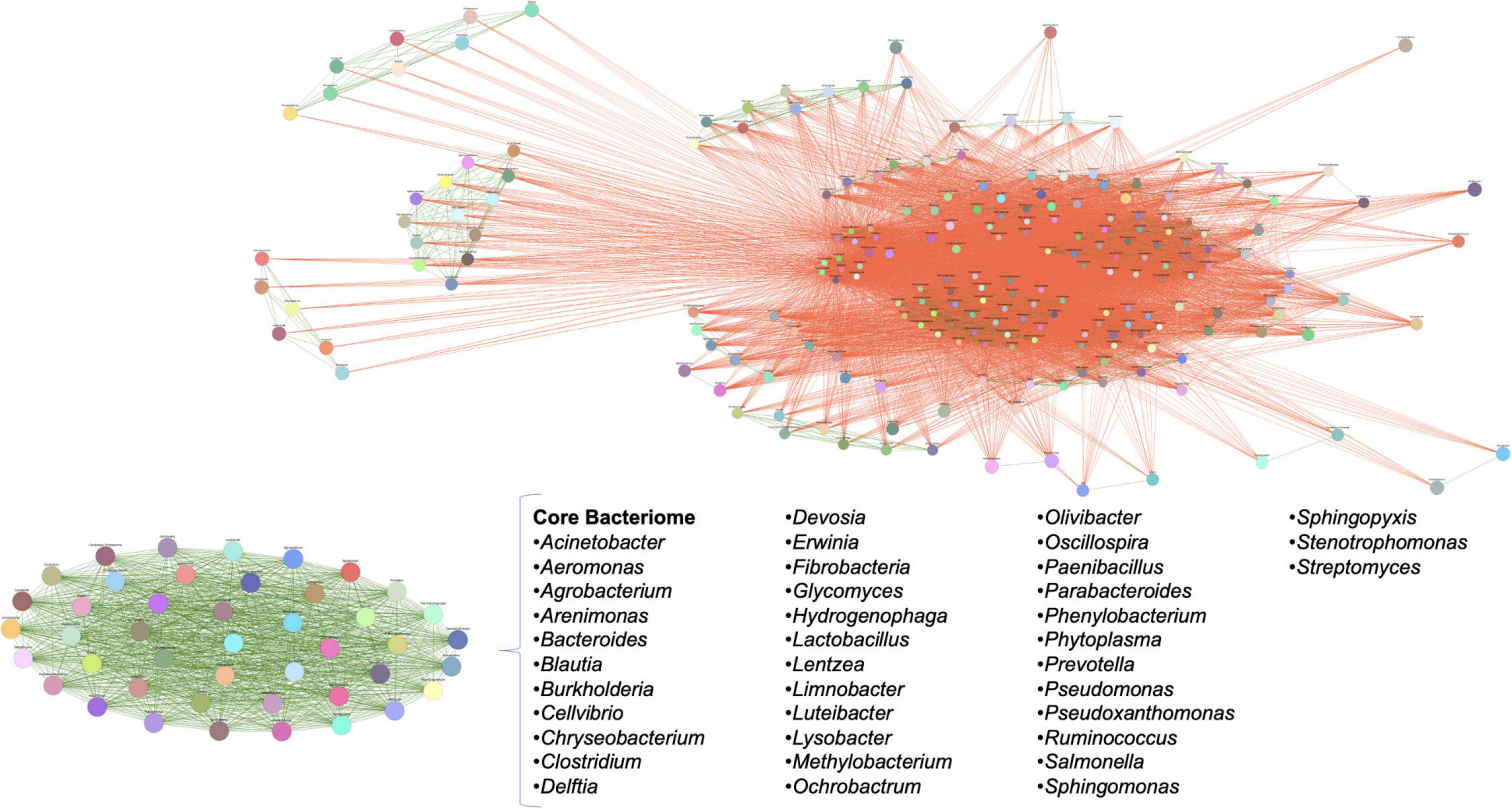
Taxonomic microbial interaction networks of representative individuals in teosinte samples. Co-occurrence diagram where green lines represent a positive correlation of bacterial genera in all analyzed samples, and red lines represent a negative correlation. The size of the circular area indicates the relative abundance of the genera. The core is shown as an oval of green lines, demonstrating that these organisms are present in all samples and can occur among them.

The analysis of the strict core bacteriome in three teosinte species and six races revealed a total of 38 genera that were present at 100% of presence with a high relative abundance (0.010% of detection) across all samples. These genera include *Acinetobacter, Aeromonas, Agrobacterium, Arenimonas, Bacteroides, Blautia, Burkholderia, Cellvibrio, Chryseobacterium, Clostridium, Delftia, Devosia, Erwinia, Fibrobacteria, Glycomyces, Hydrogenophaga, Lactobacillus, Lentzea, Limnobacter, Luteibacter, Lysobacter, Methylobacterium, Ochrobactrum, Olivibacter, Oscillospira, Paenibacillus, Parabacteroides, Phenylobacterium, Phytoplasma, Prevotella, Pseudomonas, Pseudoxanthomonas, Ruminococcus, Salmonella, Sphingomonas, Sphingopyxis, Stenotrophomonas*, and *Streptomyces*. In this same analysis, we noticed that within the negative interrelationships, at least six subgroups of between 5-15 genera are formed that share more than 80% co-occurrence, which could suggest that these genera probably also play a significant role in the specificity of each genotype in particular (**Figure 5**). Some of these genera have been found and studied in different maize samples under different techniques, which suggests the close relationship of these bacteria with maize and teosinte plants (**Table 3**).

**Table 3:** Bacterial genera associated to teosinte and maize detected in this work and other previous papers.

Bacteria detected in this work	Main sources of isolate in other works	Method of obtaining	Relevant phenotypic traits	References
*Achromobacter*	Maize root	NGS and culture	Production of siderophores	Pereira et al., 2011
*Agrobacterium*	Maize seed	NGS	Production of auxins, ACC deaminase	Walters et al., 2018
*Azospirillum*	Maize leaf/shoot	NGS and culture	Production of auxines	Cassán et al., 2009; Camilios-Neto et al., 2014
*Bacillus*	Maize rhizoplane/shoot	Culture	BFN, solubilization phosphate, production of auxins, ACC deaminase, biocontrol agent	Bacon and Hinton, 2011; Santhanam et al., 2015
*Burkholderia*	Maize shoot	Culture	BNF, production of siderophores, production of auxins, ACC deaminase, biocontrol agent	Naveed et al., 2014a
*Chitinophaga*	Maize leaf	NGS and culture	Phosphate solubilization, production of auxins, biocontrol agent	Correa-Galeote et al., 2018
*Chryseobacterium*	Maize shoot/leaf	Culture	Biocontrol agent	Lin et al., 2017
*Clostridium*	Maize/teosinte seed	NGS and culture	Solubilization phosphates	Johnston-Monje and Raizada, 2011
*Enterobacter*	Maize/teosinte seed	Culture	Biocontrol agent	Naveed et al., 2014b
*Geobacillus*	Maize rhizoplane	Culture	Biocontrol agent	Abdelkader and Esawy, 2011
*Klebsiella*	Maize shoot	Culture	BNF, phosphate solubilization	Mowafy et al., 2021
*Methylobacterium*	Maize seed	NGS	Production of auxines	Matsumura et al., 2015
*Ochrobactrum*	Maize root	NGS	Production of siderophores	Verma et al., 2022
*Pantoea*	Maize/teosinte seed/shoot	Culture	Osmotic stress tolerance	Gond et al., 2015
*Paenibacillus*	Maize seed	Culture	Biocontrol agent	Liu et al., 2016
*Pseudomonas*	Maize root	NGS and culture	Production of siderophores, production of auxins, ACC deaminase, biocontrol agent	Sandhya et al., 2017; Singh et al., 2019
*Rhizobium*	Maize rhizoplane	Culture	BFN, production of auxins, production of siderophores	Celador-Lera et al., 2017; Gao et al., 2017
*Sphingobium*	Maize shoot	Culture	Phosphate solubilization, production of auxins, production of siderophores	Pereira and Castro, 2014;
*Staphylococcus*	Maize shoot	NGS and culture	Phosphate and zinc solubilization	Marag and Suman, 2018
*Stenotrophomonas*	Maize seed/shoot	NGS and culture	Phosphate and potassium solubilization; biocontrol agent	Liu et al., 2012
*Streptomyces*	Maize rhizoplane/leaf	NGS and culture	Production of auxins, biocontrol agent	Ayswaria et al., 2020

### 3.3 Metabolic inference

The metabolic function profiles of microbial communities in teosinte seeds samples were analyzed using PICRUSt2 software and the TBtools-II v1.108 viewer. Notably, these findings are inferred through metabolic inference analysis; however, they provide valuable insights into the potential functional attributes of the microbial communities associated with different teosinte races focused on PGP and biocontrol traits. NSTI values are among 0.0011-0.0080, where *Z. mays* subsp. parviglumis (0.0076) and *Z. perennis* (0.0080) show a higher relative abundance of specific taxonomic groups than the other samples. The results show that *Z. diploperennis* harbors bacterial communities with a large number of genes related to plant growth promotion, including the carbohydrate phosphotransferase system, amino acids, sugar, and nitrogen metabolism, biosynthesis of plant hormones, proteins for enhancing seed germination and photosynthesis.

**Figure 6:**
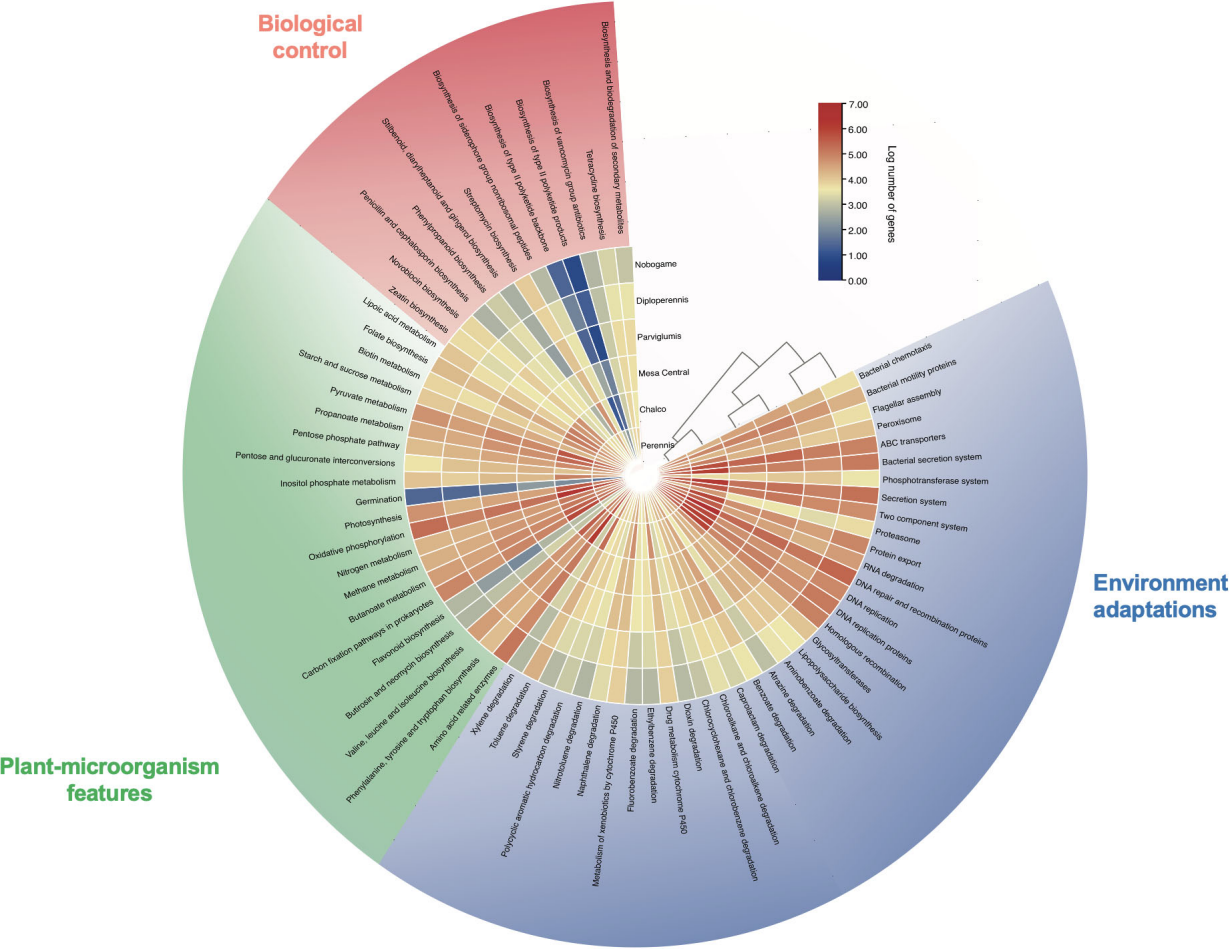
Visualization of relative gene abundance in predicted endophytic bacterial communities of teosinte seeds using a heatmap approach with PICRUSt-inferred genes. Outer semicircle, categories of environmental adaptations; inner semicircle log of the relative number of gene abundances. Some important holobiont features are biological control (pink), plant-microorganism interactions (green) and environmental adaptation (blue). The bar color gradient represents high gene abundances (red), and low (blue) abundances.

Additionally, genes responsible for the biosynthesis of biocontrol molecules, such as biosynthesis of antibiotics, antifungals, and siderophores, and genes related to adaptations to the host environment, such as chemotaxis, motility, protein export, transporters, peroxisomes, protein kinases, and degradation of recalcitrant compounds. In contrast, the *Z. mays* subsp. mexicana Chalco landrace exhibits the lowest number of genes associated with these traits, as shown in **Figure 6**.

## 4 Discussion

The diversity of bacterial communities associated with the seed of three teosinte species: *Z. diploperennis*, *Z. perennis*, and *Z. mays* subsp. mexicana races Nobogame, Balsas, Mesa Central, and Chalco were explored in this work with culture-independent methods of NGS. Only some works have addressed the issue of bacterial diversity in the teosinte endosphere from the perspective of culture-independent methods. In this sense, previous efforts focused on using bacterial DNA fingerprinting (16S rDNA TRFLP) detected a core bacteriome composed of *Clostridium*, *Paenibacillus*, and two other unidentified genera in seeds and stems of three teosinte species (Johnston-Monje & Raizada, 2011). Additionally, 18 bacteria genera were isolated and cultured from the same samples, expanding the core bacteriome of teosinte with members of the *Enterobacter*, *Methylobacterium*, *Pantoea*, and *Pseudomonas* genres. In contrast, in this work, the core bacteriome detected with NGS includes 38 bacterial genera only in seeds, confirming the presence of all previously detected but adding 36 bacteria genera for the first time. However, although the investigation of bacterial diversity is just the beginning and the comparisons between works carried out with different experimental strategies should be taken carefully, previous research using TRFLP has raised crucial questions regarding maize domestication, evolution, ethnography, geographic migration, and ecology (Johnston-Monje & Raizada, 2011; Johnston-Monje et al., 2014), all legitimate questions that can now be reconsidered with the use of NGS.

Some of the bacterial genera found in this work have been previously described as culturable endophytes in maize and teosinte plants with relevant phenotypic traits for plant-microorganism interaction, plant growth promotion, biological control, and adaptation to the environment (Chowdhury et al., 2019; Mehta et al., 2021; Wallace, 2023). However, many non-cultured bacteria genera no previously associated with teosinte and maize endophytes were also detected in the seed endosphere of teosinte, such as *Nitrospira, Scalindua*, and *Phytoplasma*, among others. These bacteria expand the potential of the teosinte microbiome for developing PGPB and biocontrol agents. The work results may be the basis for renewing efforts for isolating bacterial genera and species in specific culture media and ambiental conditions designed for those bacteria that have yet to be isolated in pure cultures.

The dynamic symbiotic relationship of endophytes with the host has essential implications for adaptation, stress tolerance, evolution, and plant domestication (Hardoim et al., 2015). Most of 38 genera of central bacteriome (core) have been recognized as PGPB, and some are also among the most abundant microorganisms found in native landrace maize samples, such as the case of *Burkholderia, Methylobacterium, Pseudomonas, Paenibacillus, Clostridium, Stenotrophomonas, Streptomyces*, and *Luteibacter* (Johnston-Monje & Raizada, 2011). As has been suggested in previous works performed with the seeds of other plants, teosinte seeds are also a vast reservoir of microorganisms of evolutionary and biotechnological interest that remain in their host despite geographic and genetic differences (Chen et al., 2018; Hamonts et al., 2018; Koskella and Bergelson, 2020; Kuźniar et al., 2020; Noble et al., 2020; Rodríguez et al., 2020). The accessory bacteriome of teosintes is possibly related to the specialization of the bacteria with their particular host. Depending on the plant host’s sampling moment, geography, or ecology, it could also be a transitory event.

The UniFrac in β-diversity analysis unveiled two clades, one that included *Z. mays* subsp. *mexicana* and another to the rest of the species and races. The association between these bacteriomes, phylogenetic proximity, and geographical distribution highlight the significance of plant genotype in influencing microbiome selection and alterations, emphasizing the role of host genetics in shaping the microbial communities associated with these plant species (Yadav et al., 2023).

The phylogeny of Mexican annual teosintes performed with microsatellite analysis recognizes two clusters *Zea mays* subsp. *mexicana* and *Z. mays* subsp. *parviglumis* on one side; meanwhile, *Z. diploperennis*, and *Z. perennis* on the other share an earlier common ancestor (Matsuoka et al., 2002; Fukunaga et al., 2005). This phylogenetic scenario could be related to the greater bacterial diversity associated with earlier teosinte species since a longer available evolutionary time to establish a symbiosis and co-evolve with free-living bacteria than the other races. However, other geographic, ecological, or evolutive scenarios cannot be discarded.

The taxonomic microbial interaction network, constructed using representative bacterial genera from the core bacteriome in teosinte samples, exhibited a complex and extensive structure. These findings indicate that the seeds of teosinte act as a “Noah’s Ark,” which possibly facilitates the vertical transmission of essential symbiotic bacteria for the survival and growth of the subsequent plant generation in new and challenging environments. When considering the assumption that the teosinte seeds originate from diverse conditions, it becomes evident that the microbial interaction network within these seeds is crucial for the plants’ adaptation and resilience. This intricate network of interactions among microbial taxa suggests a cooperative and interdependent relationship between bacteria and their plant host (Li et al., 2019; Verma & White, 2019; Bomfim et al., 2020).

By implementing caution and considering the current state of knowledge, identifying bacteria at the genus level can provide valuable insights into their phenotypic characteristics and their ability to establish symbiotic relationships with plants (Fitzpatrick et al., 2020; Liu et al., 2020; Morella et al., 2020; Trivedi et al., 2020). The outstanding similarity in the diversity and relative abundance of bacterial genera among teosintes indicates the presence of a functional and stable microbiome despite variations in recognized bacterial taxonomy. By conducting a thorough analysis of bacterial diversity and their phenotypic traits, we can better understand the role and symbiotic interactions of these bacterial communities throughout the plants’ life cycle (Berg et al., 2010; Reinhold-Hurek and Hurek, 2011; Sessitsch et al., 2012; Belimov et al., 2015; Khatabi et al., 2019).

The prediction of the functional profiles of teosinte endophytes focuses on three critical components in the plant-microorganism symbiosis: adaptation to the host environment, specific symbiotic activities, and biological control of plant pathogens. The endophyte seed bacteria of teosinte participate in the potential establishment and development of the plant holobiont through the secretion of enzymes that break down complex organic matter, allowing adequate access to nutrients and bacterial motility to enable and facilitate colonization and establishing beneficial interactions and chemical communication systems such as quorum sensing to synchronize bacteria-bacteria interaction and production of phytohormones that lead plant-bacteria communication (Vandana et al., 2021). Also, bacterial genera with a potential capacity for degradation of xenobiotic compounds commonly present in contaminated soils, such as atrazine, xylene, chloroalkanes, and polycyclic aromatic compounds, were detected. These bacteria detoxify the soil, recirculate carbon from generally recalcitrant compounds, and offer the plant an adaptative advantage during colonization and initial growth of plants (Li et al., 2012; Pandey et al., 2013; Thelusmond et al., 2016; Regar et al., 2019; Huang X. et al., 2022).

Besides, the bacterial digestion of starch, sucrose metabolism, biosynthesis of amino acids, phytohormones, and intermediate compounds of vital biochemical cycles are metabolic activities that promote the development of plants from germination to advanced phenological stages (Hunting et al., 2015; Cui et al., 2019; Rehman et al., 2019; Yuan et al., 2021; Mishra et al., 2022). Finally, the biosynthesis of antibiotic compounds such as streptomycin, cephalosporin, tetracycline, polyketides, and non-ribosomal peptides such as siderophores can function as antifungals and protect the seed before and during germination and early growth (Abbas et al., 2022; Huang B. et al., 2022; Yadav et al., 2022), although they could also interfere with the establishment of mutualistic mycorrhizae (Schrey et al., 2012).

The microbiome of other plants highlights the importance of diversity, structure, composition, and core bacteriomes for the production of essential metabolites for ecology and plant-microorganism interaction, as is the example of *Salvia miltiorrhiza* (Chen et al., 2018), *Hordeum vulgare* L. (Rahman et al., 2018), *Brassica napus* (Rybakova et al., 2017), among others.

These efforts help lay the foundations for understanding the specific interactions between plants and microorganisms from an evolutionary and ecological point of view, complementing these studies with more precise tools such as holo-omics sciences (Xu et al., 2021).

The knowledge of the bacterial diversity in the progenitor plants of modern maize can allow us to propose lines of research that will explore the domestication, evolution, ecology, and biogeography of the different races of the plant to the symbiosis-plant microorganism that will allow us to recognize the bacteria that harbor a potential to improve agricultural productivity under more environmentally friendly conditions.

## 5 Conclusion

The endophytic bacterial diversity of seed teosintes, encompassing *Z. diploperennis*, *Z. perennis*, and *Z. mays*, displays a rich array of dozens of bacterial genera, forming a strict core. In contrast, many others reside in accessory bacteriomes specific to each plant species. Numerous PGB bacterial genera have been identified, alongside several previously unassociated with maize or teosinte. However, it is essential to acknowledge that further experiments are needed to demonstrate the reproducibility of these findings. The results also suggest that teosinte seeds are a reservoir of many important culturable and non-culturable bacteria, potentially microorganisms with exciting properties in plant-microorganism interaction as plant growth promoters or bio-control agents. These results lay the groundwork for future research on the functional role of members of the core bacteriome in symbiosis and their possible biotechnological applications in the intelligent design of bioinoculants. This work is the first step toward defining holobiont, holohabitat, and holoniche as previously defined (Malard and Guisan, 2023).

## Data availability statement

The datasets presented in this study can be found in online repositories.The accesion number of the Bioproject in NCBI is PRJNA952205.

## Author contributions

ED-L-V-C and JH-G performed the DNA extraction, data analysis, and bioinformatics performances; ED-L-V-C, JH-G, LV-T, and CH-R designed and coordinated the study; ED-L-V-C wrote the first draft manuscript; ED-L-V-C, JH-G, LV-T, and CH-R contributed to the manuscript editing. All authors read and approved the final manuscript.
